# Communication on the Treatment of Chronic Gonorrhœa

**Published:** 1892-10

**Authors:** T. Trzcinski

**Affiliations:** Warsaw


### Selections and Abstracts.

---

COMMUNCIATION ON THE TREATMENT OF CHRONIC GONORRHŒA. (Beitrag zur Behandlung des chronischen Trippers. *Archiv. fur Dermatologie und Syphilis*, 11. Heft, 1892.) By Dr. T. Trzcinski, Warsaw.

This author, after careful observation of many cases, has come to agree with the statistics of Letzel, that ninety-two and a half per cent. of all gonorrhœas become posterior and last for many months in spite of any treatment. He is inclined to put but little stress upon the presence of stricture as being the prominent factor in keeping up a discharge in the more recent cases, but advises in all old cases, that the urethra should be carefully examined and all contractions be removed. As regards local treatment, he objects to the methods of Guyon and Ultzmann as being unscientific and too severe in most cases, while the application of medicaments in the form of suppositories and salves, he considers even worse. His faith in the urethroscope is exceedingly limited, and he boldly states that the theories in regard to urethral pathology presented by Grunfeld, are preposterous. The method which he advises is a modification of that of Neiser, and consists of the daily washing of the entire urethra with a solution of one to eight thousand and gradually increasing up to one to three thousand, using a small catheter with an olive tip, which is passed gently into the bladder and the solution introduced by means of a hand-syringe. The first portion of the solution, that washing the posterior urethra, flows into the bladder, the catheter being gradually withdrawn until the compressor urethræ muscle is passed, when the fluid runs out alongside of the catheter, washing the entire anterior urethra. Under this treatment the discharge disappears in a few days, leaving only "tripperfaden" (gonorrhœal threads), which may continue for some weeks. A patient may be considered cured when he can squeeze out a drop containing no pus, and when only a few "tripperfaden" exist in the morning urine alone.—[illegible]er. *Med. Mag.*, Sept. 1892.

